# Stable and exacerbation period serum cytokine and periostin levels of the five distinct phenotypes of severe asthma

**DOI:** 10.55730/1300-0144.5418

**Published:** 2021-06-12

**Authors:** Murat TÜRK, İnsu YILMAZ, Selma GÖKAHMETOĞLU, Ayşe Nedret KOÇ

**Affiliations:** 1 Division of Allergy and Clinical Immunology, Department of Chest Diseases, Erciyes University School of Medicine, Kayseri, Turkey; 2 Department of Medical Microbiology, Erciyes University School of Medicine, Kayseri, Turkey

**Keywords:** Severe asthma, asthma phenotypes, eosinophilic asthma, allergic asthma, nonallergic asthma, type-2 high asthma

## Abstract

**Background/aim:**

The differences in molecular mechanisms during a stable period and the changes in the inflammatory responses during exacerbations between distinct severe asthma phenotypes remain unclear. In this study, we aimed to characterize stable and exacerbation period serum cytokine and periostin levels of 5 different predefined severe asthma phenotypes with real-life data. Changes in the viral infection-induced exacerbations were also analyzed.

**Materials and methods:**

Serum levels of 8 cytokines and periostin were measured from the **s**era obtained from the adult patients with five different severe asthma phenotypes based on the presence/absence of aeroallergen sensitivity, peripheral eosinophilia and chronic rhinosinusitis with nasal polyposis (CRSwNP) during stable and exacerbation periods, and from the matched controls.

**Results:**

Serum IL-13, IL-25, TSLP, and periostin levels were similar between the patient and the control groups during stable and exacerbation periods. Serum IL-25 and TSLP levels, and peripheral eosinophil count and periostin level showed a strong correlation. Stable period periostin levels were significantly higher in eosinophilic patients, and eosinophilic patients without long-term systemic steroid therapy had higher IL-13 levels. Compared to stable period, exacerbation period serum periostin levels found significantly lower [5853 (2309–8427) pg/mL vs. 4479 (2766–6495) pg/mL; p = 0.05] and periostin levels were much lower in viral infection-induced exacerbations [2913 (893–4770) pg/mL vs. 7094 (4782–9596) pg/mL; p = 0.022].

**Conclusion:**

Our study showed that serum periostin levels were decreased in viral infection-induced exacerbations and increased in the presence of eosinophilia independent from atopy and it can help to differentiate eosinophilia even if the patient is under long-term systemic steroid therapy. Also, serum IL-13 levels may reflect peripheral eosinophilia in patients without long-term systemic steroid use.

## 1. Introduction

International ERS/ATS guidelines on the definition of severe asthma define severe asthma as “asthma which requires treatment with high dose inhaled corticosteroids plus a second controller (and/or systemic corticosteroids) to prevent it from becoming ‘uncontrolled’ or which remains ‘uncontrolled’ despite this therapy” [1]. Severe asthma constitutes the majority of the health-related expenses and the management of the disease remains challenging [1,2].

Severe asthma is a heterogeneous condition that has different phenotypes with distinct clinical characteristics and different endotypes with distinct underlying pathophysiological mechanisms [3]. The current personalized severe asthma treatment protocols involve targeted biological therapies based on the phenotypes and biological markers [4,5]. Hence cheap, feasible, and easy-to-access biological markers that may help to identify the asthma phenotypes and potential treatment options for these phenotypes are needed. In our clinical practice, we identify asthma phenotypes according to the peripheral eosinophilia and/or atopy status of the patients [6,7]. This classification is helpful in type-2 (T2)-high and T2-low asthma differentiation and T2 targeted biological agent choice.

The differences in molecular mechanisms between the phenotypes remain unclear and also phenotypes may not be always adequate to reveal the underlying pathophysiological processes, which are called endotypes. Two main endotypes of severe asthma are currently acknowledged: T2-high asthma, in which there is a significant T2 inflammation in airways; and T2-low asthma, where T2 inflammation is not significant [8–10]. T2-high asthma is typically characterized by eosinophilic airway inflammation of various degrees. Due to allergen-independent signaling processes, allergic mediators may not always be evident in T2-high asthma [8]. Downstream pathways are much better clarified in the T2-high endotype which is mainly regulated by IL-4, IL-5, and IL-13 producing Th2 and type-2 innate lymphoid (ILC2) cells [11].

Patients with severe asthma experience frequent exacerbations and viral infections are among the most common causes [12]. It is known that viral infections increase the expression of airway epithelium-born cytokines such as IL-25, IL-33, and thymic stromal lymphopoietin (TSLP) and stimulate ILC2 responses. T1 and/or T2 inflammatory response profiles may also emerge depending on the virus type [12]. The changes in the inflammatory response in different asthma phenotypes, particularly in viral infection-induced exacerbation periods, remain unclear. Therefore, in this study, we aimed to characterize stable and exacerbation period peripheral blood cytokine and periostin levels of 5 different predefined severe asthma phenotypes with real-life data. Changes in the viral infection-induced exacerbations were also analyzed.

## 2. Materials and methods

### 2.1. Subjects and severe asthma phenotypes

This prospective observational study was conducted in Erciyes University Division of Allergy and Clinical Immunology, Turkey, between November 2018 and October 2019. Adult patients who were under follow-up in our clinic for at least 6 months with the diagnosis of severe asthma [1] and matched control subjects were included. Inclusion and exclusion criteria are listed in Table 1. Written informed consent was obtained from all participants. The study was approved by the research and ethics committees of Erciyes University (2018/289). The study was registered to the NIH trial registry with the identifier NCT03563521.

We defined five different severe asthma phenotypes based on the presence/absence of aeroallergen sensitivity (at least one perennial aeroallergen skin prick test positivity of the 13 common aeroallergens), peripheral eosinophilia and chronic rhinosinusitis with nasal polyposis (CRSwNP) in accordance with our previously published asthma phenotyping system, which is routinely used in our clinical practice [6] (Table 2). In order to better reflect the real-life data and make the subgroup analysis possible, patients who were under long-term systemic steroid treatment were also included in the study. Matched controlled group consisted of healthy subjects without asthma, aeroallergen sensitivity, and peripheral eosinophilia.

### 2.2. Sample collection and measurements

The patients were evaluated during stable and also, if occurred, during the exacerbation periods. For the stable period data, pulmonary function data, ACT score, and venous blood samples of the patients were collected and separated at the same visit. Acute and progressive worsening of asthmatic symptoms which require the use of systemic corticosteroids or an increase in the use of daily maintenance systemic corticosteroids to prevent a serious outcome accepted as acute exacerbation, and venous blood samples were collected and separated at the same visit.

Differential white blood cell count was carried out from all venous blood samples at the same visit using a Beckman Coulter Automated Complete Blood Count Analyzer (Beckman Coulter Inc., Fullerton, Miami, FL). Serum samples were frozen at −70 °C until the day of analysis and thawed once before analysis. The serum concentrations of 8 cytokines and periostin were measured with a sandwich enzyme-linked immunosorbent assay system [IL-4 (EK0404), IL-5 (EK0407), IL-10 (EK0416), IL-13 (EK0424), IL-17 (EK0430), IL-17E/25 (EK0793), IL-33 (EK0929), TSLP (EK0958), periostin/OSF2 (EK0985) ELISA Kit PicoKine™ (Boster Biological Technology, Pleasanton CA, USA)] according to the manufacturer instructions.

### 2.3. Upper airway viral screening with multiplex PCR

In every acute exacerbation, possible triggers were evaluated. If viral upper respiratory tract infection was suspected as the etiology by the clinician, a nasopharyngeal flocked swab was obtained and cultivated in a viral liquid medium. Clinical samples were analyzed using the Fast-track Respiratory Pathogen assay (Fast-track Diagnostics, Luxembourg) on the very same day. This panel was used in a multiplex PCR assay that detects respiratory pathogens, including human respiratory syncytial viruses A/B, influenza A virus, influenza A (H1N1) virus, influenza B virus, human adenovirus, human parainfluenza virus types 1–4, human rhinovirus, human enterovirus, human metapneumovirus A/B, human bocavirus, human coronavirus types OC43, 229E, NL63, and HKU1, human parechovirus, and *Mycoplasma pneumonia*.

### 2.4. Statistical analysis

Data were analyzed using SPSS software version 17 (SPSS Inc, Chicago, Illinois, USA). Intergroup comparisons of numerical variables were made using one-way ANOVA or Kruskal-Wallis test according to the distribution and intergroup comparisons of categorical variables were made using chi-square test. Protein concentration (cytokines and periostin) data were expressed as mean [range] and a comparison of cytokine and periostin levels during stable and exacerbation periods were performed with the Wilcoxon test. P-values of less than 0.05 were considered statistically significant. Post-hoc power analysis was carried out to evaluate the minimum sample size required to achieve a power of 80% at a 5% alpha level.

## 3. Results

Ninety-one volunteers (76 patient group, 15 control group) were included in the study (Table 2). The mean age was 45.5 ± 10.4 and 77 (85%) of all volunteers were female. In the patient group, the mean peripheral eosinophil count was 316.7 (130–420) cells/mL, 36 (48%) had atopy and 19 (25%) were under long-term systemic steroid therapy (Table 3).

There was no significant difference in gender, age, FEV_1_, and ACT scores distribution among the defined severe asthma phenotypes. The control group and the patient group had similar age and gender distribution. Systemic steroid use was significantly higher in phenotype 4 (Table 3).

### 3.1. Stable period measurements

**Figure 1:**
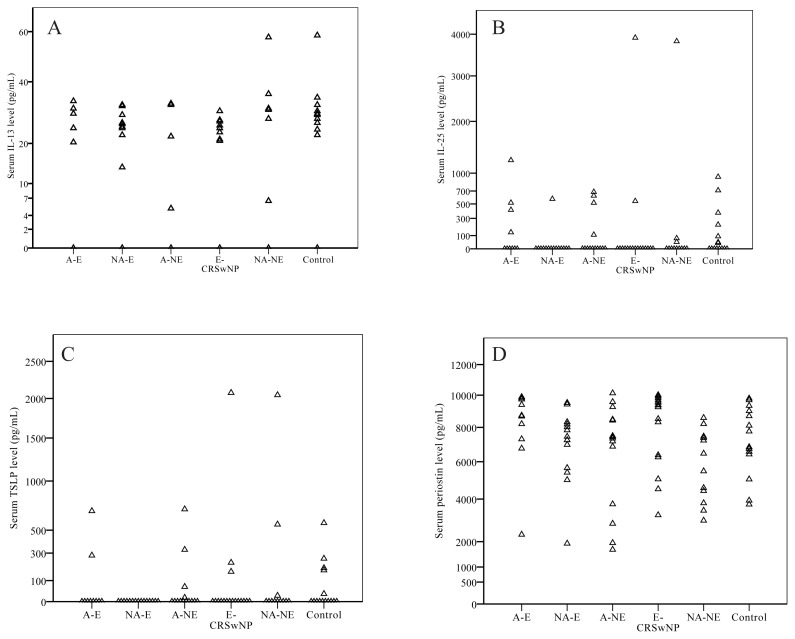
Stable period serum IL-13 (**A**), IL-25 (**B**), TSLP (**C**) and periostin (**D**) levels of five different severe asthma phenotypes and the control group determined by ELISA.

In the patient group, IL-4 was detectable in 1 patient, IL-5 in 1 patient, IL-10 in 2 patients, IL-17 in 1 patient, and IL-33 in 3 patients in the stable period. Therefore, no further comparison was done with these cytokines. Mean IL-13 level was 13.1 pg/mL (Figure 1A), IL-25 level was 194.2 pg/mL (Figure 1B), TSLP level was 105.2 pg/mL (Figure 1C) and periostin level was 7194 pg/mL (Figure 1D) in the patient group (Table 4). Serum IL-13, IL-25, TSLP, and periostin levels were similar between the patient group and the control group (p = 0.095; 0.072; 0.221 and 0.696; respectively).

#### 3.1.1. IL-13

Even though there was no significant difference in IL-13 levels between the control and the patient groups, when phenotype 3 was compared with the control group, IL-13 levels were found significantly lower in this phenotype [6.1 (0–5.2) pg/mL vs. 20.8 (0–29.9) pg/mL; p = 0.026] (Table 4). There was no difference in IL-13 levels when other asthma phenotypes were compared to the control group.

In order to find the possible effect of systemic steroid use on serum cytokine levels within different phenotypes, patients with and without long-term systemic steroid therapy were compared. We found significantly higher IL-13 levels in atopic patients under systemic steroid therapy and eosinophilic patients without systemic steroid therapy (Table 5).

There was no correlation between ACT scores, FEV_1_ values, peripheral eosinophil counts, and IL-13 levels during a stable period.

#### 3.1.2. IL-25

**Figure 2:**
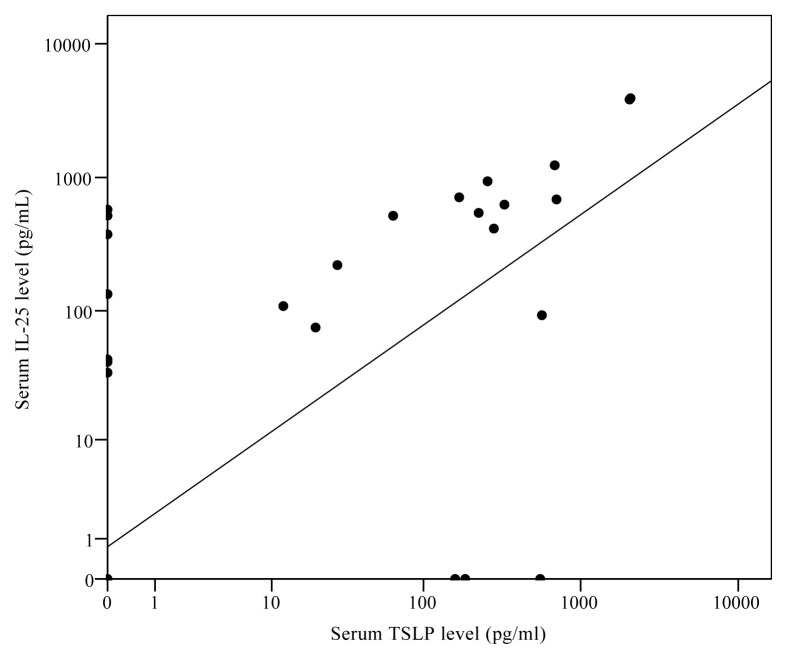
In stable period, serum levels of IL-25 and TSLP showed a strong correlation (r = 0.963; p < 0.001).

Even though there was no difference in IL-25 levels between the control and the patient groups, when phenotype 2 was compared with the control group, IL-25 levels were significantly lower in this phenotype [0 vs. 160.6 (0–220.6) pg/mL; p = 0.02] (Table 4). There was no difference in IL-25 levels when other phenotypes were compared to the control group. There was a strong correlation between IL-25 levels and TSLP levels in stable period (r = 0.963; p < 0.001) (Figure 2), no such correlation was found with the other cytokines. In patients without systemic steroid therapy, there was an almost-significant difference between the eosinophilic and noneosinophilic groups (Table 5).

#### 3.1.3. TSLP

TSLP levels were significantly lower in phenotype 2 compared to the control group [0 vs. 80.4 (0–169) pg/mL; p = 0.017] (Table 4). There was no difference in TSLP levels when other asthma phenotypes were compared to the control group. In patients under systemic steroid therapy, the difference in TSLP levels between the eosinophilic and noneosinophilic groups was significant [0 vs. 149 (0–16) pg/mL; p = 0.025] (Table 5).

#### 3.1.4. Periostin

**Figure 3:**
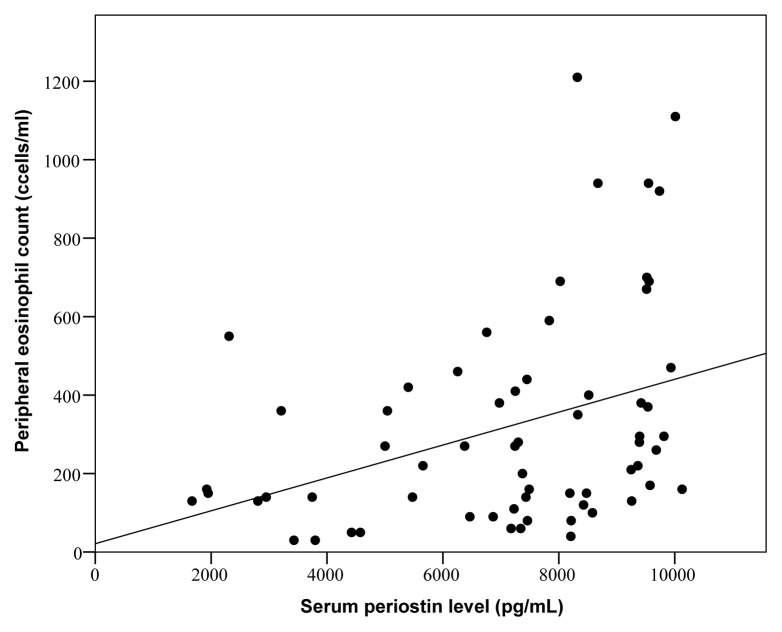
In stable period, serum periostin level and peripheral eosinophil count showed a significant correlation (r = 0.351, p = 0.004).

Periostin levels were significantly higher in phenotype 1 and phenotype 4 when compared to phenotype 5 [8067 (7028–9777) pg/mL vs. 5830 (3953–7437) pg/mL; p = 0.015 and 8137 (6315–9620) pg/mL vs. 5830 (3953–7437) pg/mL; p = 0.006, respectively] (Table 4). Stable period periostin levels were also significantly higher in eosinophilic patients [7783 (6660–9523) pg/mL vs. 6242 (3783–8267) pg/mL; p = 0.009] (Table 5). This difference was more prominent in patients without systemic steroid therapy. Peripheral eosinophil count was significantly correlated with the stable period periostin levels (r = 0.351, p = 0.004) (Figure 3). Presence of atopy had no significant effect on periostin levels.

### 3.2. Exacerbation period measurements

During the study, 23 patients had asthma exacerbation and serum samples were collected. Six of those had atopic eosinophilic, 5 had nonatopic eosinophilic, 7 had atopic noneosinophilic, 3 had CRSwNP eosinophilic and 2 had nonatopic noneosinophilic phenotype. The presence of systemic steroid therapy, atopy, or eosinophilia had no effect on exacerbation frequency.

#### 3.2.1. IL-13

There was no difference in IL-13 levels between asthma phenotypes during exacerbations (Table 6). However, IL-13 levels were significantly different between atopic and nonatopic patients [12.5 (0–13.74) pg/mL vs. 1.1 (0–3.19) pg/mL; p = 0.02]. No such difference was found for the presence of eosinophilia or systemic steroid use.

#### 3.2.2. IL-25

There was no difference in serum IL-25 levels between asthma phenotypes during exacerbations. The presence of systemic steroid therapy, atopy, or eosinophilia had no effect on IL-25 levels. There was a strong correlation between IL-25 and TSLP levels (r = 0.895, p < 0.001). No such correlation was found with the other cytokines.

#### 3.2.3. TSLP

There was no difference in serum TSLP levels between asthma phenotypes during exacerbations. The presence of systemic steroid therapy, atopy, or eosinophilia had no effect on TSLP levels.

#### 3.2.4. Periostin

There was no difference in serum periostin levels between asthma phenotypes during exacerbations. The presence of systemic steroid therapy, atopy, or eosinophilia had no effect on periostin levels.

### 3.3. Comparison of stable and exacerbation periods

**Figure 4:**
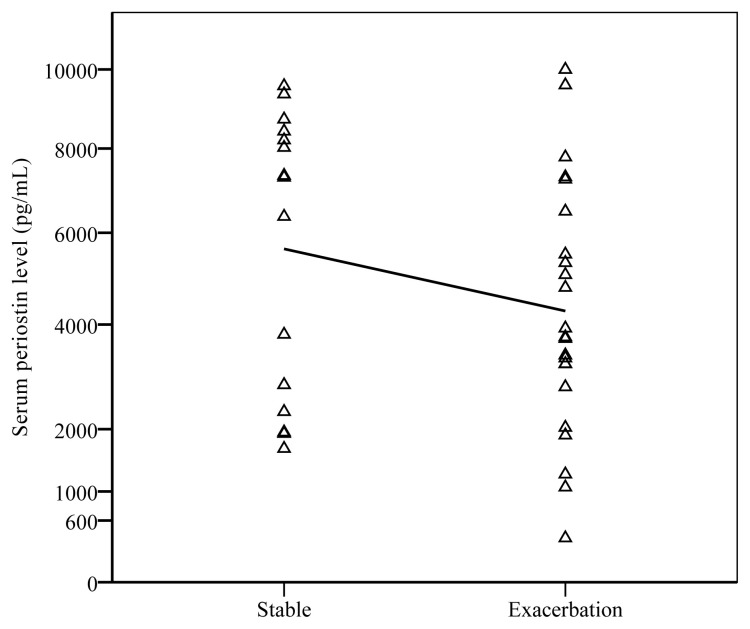
Serum periostin levels were compared between stable and exacerbation periods.

When IL-13, IL-25, TSLP, and periostin levels during the stable and exacerbation periods were compared, 23 patients with exacerbation had significantly lower periostin levels during the exacerbation period [5853 (2309–8427) pg/mL vs. 4479 (2766–6495) pg/mL; p = 0.05] (Figure 4). No such change was depicted with the other cytokines (Table 7). All these 23 patients had increased peripheral eosinophil counts during exacerbations [229 (120–280) cells/mL for stable and 780 (130–490) cells/mL for exacerbation; p = 0.009]. Periostin levels and blood eosinophil counts were also correlated during exacerbations (r = 0.454; p = 0.029).

### 3.4. Change in viral exacerbations

Thirteen patients had a nasopharyngeal mucosal swab taken due to upper respiratory tract infection suspicion during the exacerbation period. In 6 of these, viral etiology was detected by multiplex PCR (2 RSV, 2 influenza A, 1 influenza B, and 1 rhinovirus). Periostin levels were significantly lower in virus-positive group compared to virus-negative group during exacerbation period [2913 (893–4770) pg/mL vs. 7094 (4782–9596) pg/mL; p = 0.022]. No such difference was present with the other cytokines. A post hoc power analysis indicated low power (power < 80%) to detect differences between the patient and control group.

## 4. Discussion

In the present study, in 5 distinct clinic/inflammatory severe asthma phenotypes which were defined based on the presence or absence of atopy, peripheral eosinophilia, and CRSwNP in the real-world settings, serum levels of 8 different cytokines and periostin were studied during stable and exacerbation periods. Even though serum IL-13, IL-25, TSLP, and periostin levels showed no significant difference between the patient and control groups, each serum protein showed a significant difference among asthma phenotypes according to the presence of atopy, eosinophilia, exacerbation, viral infection, or use of long-term systemic steroids.

Asthma is a heterogeneous disease which includes different clinical phenotypes and distinct pathophysiological endotypes. T2-high phenotype constitutes approximately 50%–70% of all asthma patients [5]. IL-4, IL-5, and IL-13 are the main cytokines involved in T2 inflammation. In our study, only serum IL-13 showed significance out of these three cytokines. IL-13 is a central effector cytokine in asthma and the pivotal regulator in IgE synthesis, goblet cell hyperplasia, airway remodeling, mucus hypersecretion, and airway hyperresponsiveness [13]. IL-13 is also a central inducer of periostin production from airway epithelial cells [14]. Since the key mediator role of IL-13 is evident in allergic inflammation, measurement of IL-13 levels with direct or indirect markers is important in the diagnosis and endotyping of severe asthma. IL-13 can be measured in induced sputum; however, sputum induction and interpretation are not feasible in real-life. As in most the cytokines, a very little amount of IL-13 passes to the bloodstream and usually is very hard to depict in the serum. Since our study involves severe asthma patients with possibly a more severe type-2 inflammation and a higher amount of circulating cytokines, we tried to measure IL-13 levels directly in the serum. We found no significant difference in serum IL-13 levels between the patient and the control groups. When compared in pairs, IL-13 levels were significantly lower in phenotype 3 in our study, in which none of the patients were under long-term systemic steroid treatment. We believe this finding is most likely due to the small percentage of IL-13 positivity in phenotype 3, since IL-13 levels in phenotype 3 were found significantly lower even than in phenotype 5 (nonatopic, noneosinophilic severe asthma phenotype). IL-13 levels were also significantly higher in atopic patients under long-term systemic steroid therapy and eosinophilic patients without systemic steroid therapy. Eighteen of 19 patients under systemic steroid therapy also had peripheral eosinophilia, and 13 were in the phenotype 4 group. Therefore, we may suggest that IL-13 levels may be affected by peripheral eosinophilia rather than the presence of atopy. It was reported that IL-13 can induce eosinophil activation, recruitment and prolongs eosinophil survival [15]. IL-4 and IL-13 are both potent inducers of VCAM-1 in endothelial cells, which are important for the recruitment of eosinophils [13]. The relationship between serum IL-13 levels and atopic-eosinophilic asthma has been reported in previous studies [16–19]. Hussein et al. showed a higher serum IL-13 levels in children with atopic asthma compared to the control group and a correlation between serum IL-13 levels and disease severity [17]. Peripheral eosinophil counts were particularly high in this study and as for IL-13, peripheral eosinophilia was also correlated with disease severity. Kalinauskaite-Zukauske et al. reported significantly higher IL-13 levels in atopic asthma patients compared to the control group and an increase in IL-13 levels after the bronchial allergen challenge test with *D. pteronyssinus* [18]. Basal peripheral eosinophil counts were near twice the control group in atopic asthmatic patients in this study. In their clustering study using multidimensional endotyping with different biomarkers and clinical phenotypes, Agache et al. found that serum IL-13 is a reliable biomarker to detect peripheral eosinophilia and, in another study, they also showed that high eosinophilic moderate asthma cluster had significantly increased serum IL-13 levels [20, 21]. Apart from asthma, serum IL-13 levels are also shown to increase in CRSwNP. In their study where serum cytokine levels were compared in chronic rhinosinusitis with nasal polyps and control group, Nabavi et al. reported a significantly higher serum IL-13 level in CRSwNP group. The authors also showed that IL-13 levels were not affected by atopy status [22].

Allergens, toxic substances, and viral infections cause the release of IL-25, IL-33, TSLP cytokines, the so-called alarmins, from the airway epithelium and induce type-2 inflammation via ILC2 and Th2 cells in asthma [23]. IL-33 could not be detected in serum in our study. There was also no difference in IL-25 and TSLP levels between the patient and control groups. IL-25 and TSLP were significantly correlated in both stable and exacerbation periods. Their levels were also significantly lower in patients with peripheral eosinophilia without long-term systemic steroid therapy. In a previous study where serum levels of 24 different cytokines and chemokines were studied in severe asthmatic patients, IL-25 levels were also similar in control and asthma groups [16]. In addition, TSLP levels were also significantly higher in asthma groups. The authors also stated that there was no difference in these two cytokines when controlled and uncontrolled asthma patients were compared. In another study, where baseline IL-25 and TSLP levels were similar between atopic asthmatic patients and the control group, a significant increase in these two cytokine levels was observed after the bronchial inhaler challenge [18]. In our study, IL-25 and TSLP were not detected in the serum of phenotype 2 patients in which none of the patients were under long-term systemic steroid therapy. These two cytokines could not be detected in phenotypes 2 and 4, where eosinophilia is prominent, but had significant or almost-significantly higher levels in phenotypes 1 and 3, where atopy is present when compared to phenotype 2. Therefore, we may speculate that the serum positivity of these cytokines may be more valuable for demonstrating the presence of atopy.

Periostin is a matricellular protein broadly secreted by many tissues including the airway epithelium, musculoskeletal system, and gastrointestinal tract. It is upregulated by type-2 cytokines like IL-4 and IL-13 and correlates with other type-2 biomarkers such as FeNO, sputum eosinophilia, blood eosinophilia, and total IgE [24, 25]. In concordance with our results, recent studies showed that serum periostin measurement may be inadequate in differentiating asthmatic and healthy subjects [14, 16, 25]. Serum periostin levels were reported to be higher even in professional athletes compared to asthmatic patients [26]. Periostin is pronounced to be more useful in the evaluation of response to monoclonal antibodies and systemic steroid therapy, rather than asthma diagnosis. In addition, it may also be used in differentiating asthma subtypes where airway eosinophilia is present [14, 27]. Serum periostin levels were elevated in asthmatics with CRSwNP and serum periostin could distinguish these patients from asthmatics without any comorbidities [28]. In our study, we found that periostin levels were strongly correlated with peripheral eosinophilia levels and could be helpful in differentiating eosinophilia despite systemic steroid use. Agache et al. also showed that serum periostin is one of the best predictors of blood eosinophilia [20]. In addition to its inadequacy for asthma diagnosis, there is also very limited data on its use during exacerbations. Semprini et al. recorded weekly serum periostin levels for 12 weeks in patients who received systemic steroid therapy after exacerbations. The authors stated that serum periostin levels varied during this period and 12^th^-week levels were higher than baseline levels at admission. The difference showed a clear tendency to significance [median 3.93 (3.87–4.16) ng/mL vs. 3.89 (3.74–4.2) ng/mL; p = 0.06] [29]. However, exacerbation etiologies were not included for comparison in this study. It is of great importance that this and future studies investigate periostin level changes according to exacerbation etiologies, even according to different respiratory tract pathogens.

Viral infections are frequent triggers of asthma exacerbations. Influenza, coronavirus, parainfluenza, and most commonly rhinovirus are associated with exacerbations of asthma in children and adults [30]. It has been shown that viral infections may interact with the allergic inflammation in the airways and induce exacerbations in asthmatic patients. Allergic sensitization or eosinophilic inflammation further increases the risk for wheezing illnesses [30]. Following the local epithelial damage due to viral infection, production of IL-25, IL-33, and TSLP increases, which stimulates ILC2s, and as a result, T2 inflammation is induced. On the other hand, in non-T2 asthma models, after epithelial injury, IL-6, TNF, and IL-1a production are stimulated, and neutrophilic inflammation is induced [12]. There is evidence that IgE-mediated allergic inflammation could reduce virus-induced interferon responses in asthma [30, 31]. In contrast, it was also shown that type-1 interferons can inhibit Th2 immune responses [32]. Pritchard et al. showed that IFN-alpha and IFN-B reduce Th2 cytokine production and interestingly they also found that when the activity of these interferons was blocked, IL-13 secretion was increased [32]. The inhibitory effect of interferons on type 2 cytokine response has also been shown in other studies. It was shown that IFN-alpha can markedly inhibit IL-5 production [33]. Type-1 interferons can also block Th2 cytokine secretion through the inhibition of GATA3 [34]. Supporting these findings, in our study, during exacerbation periods serum periostin levels were found significantly lower in patients with positive viral PCR.

Our study has some limitations. First of all, the total number of patients and the number of patients in some phenotypes caused low power for comparisons. Even though we had similar results, the number of patients in the exacerbation period and patients with positive viral PCR were lower than expected. Secondly, we only performed multiplex PCR in cases with clinical suspicion of upper respiratory tract infections. This might cause selection bias. Thirdly, some of the cytokines could not be detected in the serum. We believe this is unrelated to a technical error since all tests were performed with the same brand assays and the same method on the serum samples that were kept in the same environment. Fourthly, in order to make a clear distinction between the predefined phenotypes (particularly phenotypes 2 and 3), we had to partially move away from real-life and exclude the patients with peripheral eosinophil count 150–300 cells/mL from the study. Another possible reason for this is that our peripheral eosinophil count cut-off value may not be able to reflect the tissue eosinophilia. Even though peripheral eosinophil count > 300 cells/mL is a strong predictor of sputum eosinophilia, and indirectly tissue eosinophilia, there is still a chance of tissue eosinophilia in the so-called noneosinophilic patients who had peripheral eosinophil count less than 150 eosinophils/mL. Further studies where eosinophilia is shown in the lower respiratory tract samples or secretions will enable more accurate results. Lastly, we studied the serum protein profiles of the phenotypes, but advanced data analytic approaches such as principal component analysis or topological data analysis were not used in contrast to the previous studies.

In conclusion, we showed that IL-13 can be depicted in the serum of severe asthmatic patients and may reflect peripheral eosinophilia in patients without systemic steroid use. In addition, serum periostin levels were increased in the presence of eosinophilia independent from atopy and it can help to differentiate eosinophilia even if the patient is under systemic steroid therapy. Further studies with larger series, which investigate whether the lower periostin levels during exacerbations could predict a viral infection as the underlying etiology and studies on the variations of periostin levels during exacerbations secondary to viral infections are needed. Since IL-4, IL-5, IL-10, IL-17, and IL-33 would not be measured in peripheral blood samples, we think it is not feasible to use these cytokines in clinical practice or in the research of the underlying mechanisms of the asthma phenotypes. We believe our results may shine a light on severe asthma characterization and personalized medicine approaches.

## Figures and Tables

**Table 1:** Inclusion and exclusion criteria for the patients.

**Inclusion criteria**

1. Diagnosis of severe asthma according to the International ERS/ATS guideline (1)

2. Under follow-up for severe asthma for at least 6 months in our clinic

3. Meets the defined phenotype criteria of the study

**Exclusion criteria**

1. Under 18 years of age

2. Smoking history in the last 1 year

3. Under monoclonal antibody treatment for severe asthma or any other disease.

4. Comorbidities: malignancy, collagen tissue disease, hyperthyroidism, cardiovascular diseases, type 2 diabetes, active liver disease, acute kidney failure or any autoimmune disorder

5. Solid organ transplantation

6. Pregnancy

7. Pulmonary diseases other than asthma: chronic obstructive pulmonary disease, bronchiectasia, interstitial lung diseases, pulmonary thromboemboli

8. For the stable period assessment:
a. Asthma Control Test (ACT) < 16
b. Upper respiratory infection within the last 1 month
c. Exacerbation and/or systemic steroid treatment within the last 1 month

9. For the exacerbation period assessment:
d. Out of routine daily systemic steroid use before admission

10. Atopy with only seasonal allergen sensitivity

**Table 2:** Definition criteria of the five different severe asthma phenotypes.

Phenotypes	Definition criteria	# of volunteers
**Phenotype 1:** Atopic, eosinophilic severe asthma (A-E)§	□ At least 1 perennial aeroallergen sensitivity □ Peripheral eosinophil count > 300 cells/mL in at least 2 blood samples 1 month apart¥	11
**Phenotype 2:** Nonatopic, eosinophilic severe asthma (NA-E)	□ No aeroallergen sensitivity □ Peripheral eosinophil count > 300 cells/mL in at least 2 blood samples 1 month apart¥	18
**Phenotype 3:** Atopic, noneosinophilic severe asthma (A-NE)§	□ At least 1 perennial aeroallergen sensitivity □ Peripheral eosinophil count < 150 cells/mL in at least 2 blood samples 1 month apart during steroid-naive period	16
**Phenotype 4:** Eosinophilic severe asthma with comorbid chronic rhinosinusitis with nasal polyposis (E-CRSwNP)§	□ Chronic rhinosinusitis and nasal polyposis diagnosis by physical examination, nasal endoscopy or PNCT □ NERD may accompany □ Aeroallergen sensitivity may accompany □ Peripheral eosinophil count > 300 cells/mL in at least 2 blood samples 1 month apart¥	18
**Phenotype 5:** Nonatopic, noneosinophilic severe asthma (NA-NE)	□ No aeroallergen sensitivity □ Peripheral eosinophil count < 150 cells/mL in at least 2 blood samples 1 month apart during steroid-naive period	13
**Control group**	□ Healthy subjects without proven asthma, aeroallergen sensitivity and peripheral eosinophilia	15

PNCT: Paranasal sinus computed tomography; NERD: Nonsteroidal antiinflammatory drug exacerbated respiratory disease

§ If pollen sensitivity is present in addition to perennial allergen sensitivity, serum samples were collected out of the pollen season for stable period measurements.

¥ In case of long-term systemic steroid treatment, inclusion criteria for blood eosinophil count were accepted as > 150 cells/mL.

**Table 3:** General characteristics of the phenotypes.

	Patient group n = 76	Phenotype 1 (A-E) n = 11	Phenotype 2 (NA-E) n = 18	Phenotype 3 (A-NE) n = 16	Phenotype 4 (E-CRSwNP) n = 18	Phenotype 5 (NA-NE) n = 13	*p*
**Female gender; n (%)**	66 (87)	9 (82)	15 (83)	14 (88)	16 (89)	12 (92)	0.93
**Age (years); mean ± SD**	45.6 ± 11.1	46 ± 11.7	47.4 ± 12.8	41 ± 11.9	48.1 ± 8.8	46.4 ± 9.9	0.466
**Chronic rhinosinusitis; n (%)**	52 (70)	6 (60)	8 (44)	14 (93)	18 (100)	6 (46)	<0.001
**Long-term systemic steroid use; n (%)**	19 (25)	1 (9)	3 (17)	2 (13)	13 (72)	0	<0.001
**FEV** **_1_** **; mean % of predicted ± SD**	90.5 ± 20.1	84.9 ± 17	93.1 ± 27.1	95.3 ± 24.3	84.8 ± 15.7	93.3 ± 11.5	0.578
**FEV** **_1_** **; mean cc (IQR)**	2451 (1860–2620)	2441 (1715–3020)	2384 (1830–2707)	2738 (2100–3370)	2197 (1645–2545)	2583 (2227–2572)	0.488
**ACT; mean (IQR)**	20.8 (20–23)	20.9 (20–23)	20.9 (20–23)	19 (18–22)	22 (20–24)	21.1 (20–22)	0.183
**Peripheral eosinophil count; mean % (IQR)**	3.7 (1.5–4.8)	6.2 (3.8–8.1)	5 (3.1–7.8)	1.8 (1.3–2.5)	4.8 (2.3–6)	1.26 (0.73–1.68)	<0.001
**Peripheral eosinophil count; mean cells/mL (IQR)**	316 (130–420)	548 (295–920)	408 (270–552)	122 (90–150)	465.9 (240–580)	91.7 (50–132)	<0.001

SD: standard deviation; FEV_1_: forced expiratory volume in 1 s; ACT: asthma control test; IQR: interquartile range

**Table 4:** Comparison of IL-13, IL-25, TSLP, and periostin levels in asthma phenotypes and control group.

	Patient group n=76	Phenotype 1 (A-E)	Phenotype 2 (NA-E)	Phenotype 3 (A-NE)	Phenotype 4 (E-CRSwNP)	Phenotype 5 (NA-NE)	Control group	*p* *
**IL-13; mean pg/mL (IQR)**	13.1 (0–26.1)	15.3 (0–29.9)	17 (0–26.1)	6.1 (0–5.2)	12.9 (0–24.89)	15.7 (0–30.59)	20.8 (0–29.9)	0.215
**IL-25; mean pg/mL (IQR)**	194.2 (0)	240.6 (0–466)	0	138 (0–133)	0	328.6 (0–32.03)	160.6 (0–220.6)	0.196
**TSLP; mean pg/mL (IQR)**	105.2 (0)	107.3 (0–140)	0	73.8 (0–12)	0	218.2 (0–14.9)	80.4 (0–169)	0.353
**Periostin; mean pg/mL (IQR)**	7194 (5520–9385)	8067 (7028–9777)	7182 (5655–8328)	6703 (3744–8478)	8137 (6315–9620)	5830 (3953–7437)	7217 (6422–9010)	0.041

* Comparison of all phenotypes and the control group (Kruskal Wallis test).

**Table 5:** Effects of systemic steroid therapy, atopy and peripheral eosinophilia on stable period IL-13, IL-25, TSLP, and periostin levels.

n = 76		Atopy			Peripheral eosinophilia		
		With	Without	*p*	With	Without	*p*
**With long term systemic steroid use n = 19**	**IL-13; mean pg/mL (IQR)**	19.2 (0–30)	5.9 (0–15.6)	0.033	n/a*		
	**IL-25; mean pg/mL (IQR)**	527.8 (0–408)	139.8 (0–402.3)	0.736			
	**TSLP; mean pg/mL (IQR)**	292 (0–290)	0	0.36			
	**Periostin; mean pg/mL (IQR)**	8284 (7749–9615)	6719 (3535–9153)	0.183			
**Without long term systemic steroid use n = 57**	**IL-13; mean pg/mL (IQR)**	7.2 (0–20.7)	17.2 (0–29.9)	0.019	17.2 (0–28)	8.6 (0–14.3)	0.034
	**IL-25; mean pg/mL (IQR)**	130.4 (0–185)	0	0.145	0	235.2 (0–58.7)	0.081
	**TSLP; mean pg/mL (IQR)**	63.1 (0–3)	0	0.347	0	149 (0–16)	0.025
	**Periostin; mean pg/mL (IQR)**	7292 (6630–9440)	6877 (5301–8391)	0.311	8071 (7042–9518)	6153 (3770–7851)	0.007

* There was only 1 patient in the noneosinophilic group and no further comparison was done.

**Table 6:** Comparison of IL-13, IL-25, TSLP, and periostin levels between asthma phenotypes during exacerbations.

	Patient group n = 23	Phenotype 1 (A-E)	Phenotype 2 (NA-E)	Phenotype 3 (A-NE)	Phenotype 4 (E-CRSwNP)	Phenotype 5 (NA-NE)	*p*
**IL-13; mean pg/mL (IQR)**	8.5 (0.11)	18.7 (0.44.7)	1.7 (0–4.3)	10.4 (1.5–19.8)	3.4 (0–10.1)	0	0.203
**IL-25; mean pg/mL (IQR)**	300.9 (0–356)	871.4 (0.2179)	186.6 (0–466.5)	187 (0–387.5)	45 (0–135)	0	0.685
**TSLP; mean pg/mL (IQR)**	100.9 (0)	349.2 (0–873)	0	71.7 (0–176.3)	0	0	0.328
**Periostin; mean pg/mL (IQR)**	4479 (2766–6495)	3977 (1963–6290)	4231 (1161–7756)	4779 (3720–6137)	5191 (2766–9596)	4087 (385–7789)	0.926

**Table 7:** Comparison of cytokine levels during stable and exacerbation periods.

n = 23	Stable period	Exacerbation period	*p*
**IL-13; mean pg/mL (IQR)**	9.7 (0–26.5)	8.5 (0–11)	0.638
**IL-25; mean pg/mL (IQR)**	290.1 (0–575)	301 (0–356)	0.386
**TSLP; mean pg/mL (IQR)**	119.9 (0–63.8)	100.9 (0)	0.866
**Periostin; mean pg/mL (IQR)**	5853 (2309–8427)	4479 (2766–6495)	0.05
